# DNA sequencing for microbial surveillance in cystic fibrosis airways: advances, challenges, and clinical translation

**DOI:** 10.1128/cmr.00352-25

**Published:** 2026-08-03

**Authors:** Jessica A. P. Carlson-Jones, Thomas R. Goddard, Bhavya Papudeshi, Vijini Mallawaarachchi, Katrine L. Whiteson, Morgyn S. Warner, Judith M. Morton, Hubertus P. A. Jersmann, Robert A. Edwards

**Affiliations:** 1 Flinders Accelerator for Microbiome Exploration, College of Science and Engineering, Flinders University 117627, Adelaide, South Australia, Australia; 2 Department of Respiratory and Sleep Medicine, Women’s and Children’s Hospital 6053, Adelaide, South Australia, Australia; 3 College of Medicine and Public Health, Flinders University 198094, Adelaide, South Australia, Australia; 4 Faculty of Health and Medical Sciences, Adelaide Medical School, University of Adelaide 849005 , Adelaide, South Australia, Australia; 5 Department of Molecular Biology and Biochemistry, University of California Irvine 209685, Irvine, California, USA; 6 Microbiology & Infectious Diseases Directorate, SA Pathology 10107, Adelaide, South Australia, Australia; 7 Department of Thoracic Medicine, Royal Adelaide Hospital 1062, Adelaide, South Australia, Australia; Mayo Clinic Minnesota, Rochester, Minnesota, USA

**Keywords:** cystic fibrosis, metagenomics, DNA sequencing, respiratory pathogens, diagnostics

## Abstract

DNA sequencing has revolutionized microbial surveillance in cystic fibrosis (CF), transforming pathogen identification from culture-dependent to total microbial community identification using molecular-based approaches. Techniques such as 16S rRNA gene sequencing have uncovered the complexity of the CF airway microbiome, while shotgun metagenomics, metatranscriptomics, and viromics now provide strain-level, functional, and viral insights beyond bacterial identification. Despite these advances, key technical and logistical challenges remain, including the processing of high-viscosity sputum samples, overwhelming host DNA contamination, managing large data sets, and the integration of complex bioinformatic outputs into clinical workflows. Emerging innovations such as host DNA depletion protocols, targeted enrichment panels, and adaptive sampling on Oxford Nanopore platforms are helping to overcome these barriers, improving microbial recovery and sequencing efficiency. As cystic fibrosis transmembrane conductance regulator (CFTR) modulator therapies are changing the lives of people with cystic fibrosis (pwCF), sequencing offers an unprecedented opportunity to track potential microbial adaptation in response. This review investigates current advances, limitations, and translational opportunities in DNA sequencing for CF airway microbiome surveillance, highlighting how these technologies can help reshape research and clinical microbiology in the post-modulator era.

## INTRODUCTION

Cystic fibrosis (CF) is a genetic disorder caused by mutations in the cystic fibrosis transmembrane conductance regulator (CFTR) gene, resulting in defective chloride and bicarbonate ion transport across epithelial surfaces (1, 2). In the lungs, impaired CFTR function leads to dehydrated, thick, viscous mucus that impairs mucociliary clearance. This creates an environment receptive to chronic infection and inflammation, which ultimately results in progressive airway damage (3–5). Persistent microbial colonization and recurrent pulmonary exacerbations remain the primary causes of morbidity and mortality in people with CF (pwCF) (6). Therefore, identifying and understanding the pathogens and microbial communities that contribute to these infections are critical for improving disease management and clinical outcomes for pwCF (7).

Traditionally, pathogen identification in CF relies on targeted culturing of known organisms such as *Pseudomonas aeruginosa*, *Staphylococcus aureus*, *Haemophilus influenzae*, *Burkholderia cepacia* complex, and *Stenotrophomonas maltophilia* (8, 9). This gold-standard approach uses selective media and growth conditions optimized for specific pathogens of interest, enabling pathology clinics the ability to provide clinicians a report on the presence or absence of specific microorganisms and, in some cases, perform limited antimicrobial susceptibility testing. However, culture-based diagnostics detect only culturable and pre-selected organisms, overlooking the polymicrobial nature of CF airway infections. These methods underestimate community diversity and fail to capture interspecies interactions, functional profiles, and the roles that commensal or anaerobic bacteria, fungi, and viruses play in disease progression.

The advancement of DNA sequencing has revolutionized microbial surveillance, not only in CF, but also in other microbial-driven infections. This has provided researchers with the ability to utilize culture-independent methods to characterize the entire microbial communities present within a sample. Techniques such as polymerase chain reaction (PCR), 16S rRNA gene amplicon sequencing, shotgun metagenomics, and long-read sequencing now provide comprehensive insights into bacterial, viral, and fungal populations. These methods reveal not only the composition and diversity of the airway microbiome but also strain-level variation, antimicrobial resistance (AMR) genes, virulence determinants, and microbial metabolic functions. As sequencing technologies evolve, they hold increasing potential for real-time pathogen surveillance, personalized antimicrobial therapy, and improved prognostication in CF clinical care and other microbial-driven conditions.

We are at a pivotal moment in CF history with the introduction of CFTR triple modulator therapies, elexacaftor/tezacaftor/ivacaftor (ETI) and vanzacaftor/tezacaftor/deutivacaftor (VTD). The combination of correctors and potentiators used in these therapies restores CFTR protein function by improving chloride and bicarbonate transport, re-establishing salt and water balance in the airways to closely resemble that of someone without CF. The resulting improvement in mucus hydration and mucociliary clearance has transformed clinical outcomes and, ultimately, the lives of many pwCF (10–21). However, while triple modulator therapies have markedly reduced the frequency and severity of respiratory infections, pwCF still remain at risk (13, 22–24). Whether these infections involve pathogens that predominated before modulator therapy or reflect a new post-modulator microbial profile is being investigated (24–33). With advances in DNA sequencing, we now have the tools to comprehensively re-examine the CF airway microbiome and assess whether modulator therapy has reshaped or shifted its composition and function. Understanding these dynamic changes is essential for predicting long-term outcomes, guiding infection management, and informing diagnostic strategies for pwCF in the modulator era.

Despite these advances, significant technical challenges continue to limit the routine application of DNA sequencing in clinical microbiology. The high viscosity of sputum impedes efficient microbial DNA extraction, while the overwhelming abundance of human host DNA often dominates sequencing profiles, obscuring microbial reads and biasing community profiles. Overcoming these challenges is critical to achieving accurate, reproducible, and clinically meaningful metagenomic analyses. In this review, we examine recent advances in DNA sequencing technologies for microbial surveillance in CF airways, highlighting the experimental, computational, and translational challenges that currently hinder its implementation in clinical practice. We also explore emerging approaches to improve sample processing and host DNA depletion, and discuss how these innovations are paving the way toward the integration of sequencing-based microbiology into clinical care.

## ADVANCES IN DNA SEQUENCING FOR MICROBIAL SURVEILLANCE IN CYSTIC FIBROSIS AIRWAYS

**Fig 1:**
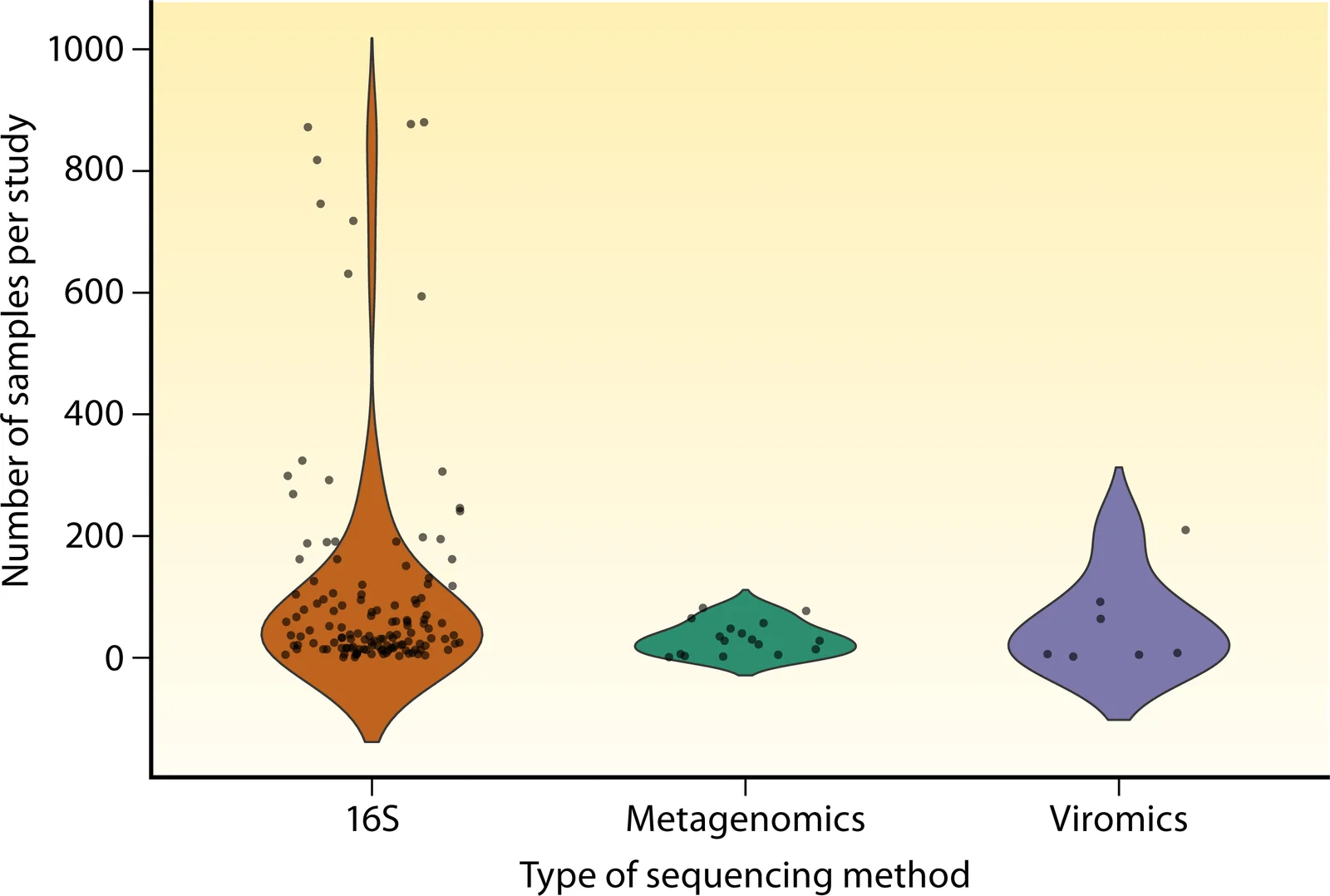
Number of samples per study for microbiome comparisons of sputum samples from pwCF sequenced by 16S amplicon sequencing, shotgun metagenomics/metatranscriptomics sequencing, or viromics. The figure was generated by the authors from a manually curated survey of cystic fibrosis microbiome sequencing studies represented in NCBI BioProjects and associated publications. Reported sample numbers, sample type, study design, and sequencing approaches were extracted where available from BioProject metadata and corresponding manuscripts. Studies without confidently assignable sample numbers were excluded from quantitative summaries. Data visualization was performed in Python using the seaborn plotting library. The figure was enhanced using Adobe Illustrator.

The advent of DNA sequencing has transformed our understanding of the airway microbiome in pwCF, shifting diagnostic paradigms from culture-dependent to molecular-based approaches (34). Polymerase chain reaction (PCR) has long been used to identify bacteria colonizing the CF lung by differential PCR using primers for specific pathogens (35), by cloning individual 16S genes into plasmids and hybridizing them with known sequences (36), by culturing individual bacteria and sequencing their 16S genes (37), and culminating with culture-independent amplicon sequencing (38). Amplicon sequencing is cheap and rapid. Currently, 16S rRNA gene amplicon sequencing is the most widely applied method for taxonomic profiling of bacterial communities in sputum samples from pwCF (Fig. 1). We found 132 data sets in the NCBI BioProjects database that employ sputum 16S sequencing to identify microbes in CF lungs, with sample sizes ranging broadly (mean = 135, median = 75); however, only 50 (38%) studies exceed 100 samples. The most extensive collection to date analyzed 880 samples (39).

**Fig 2:**
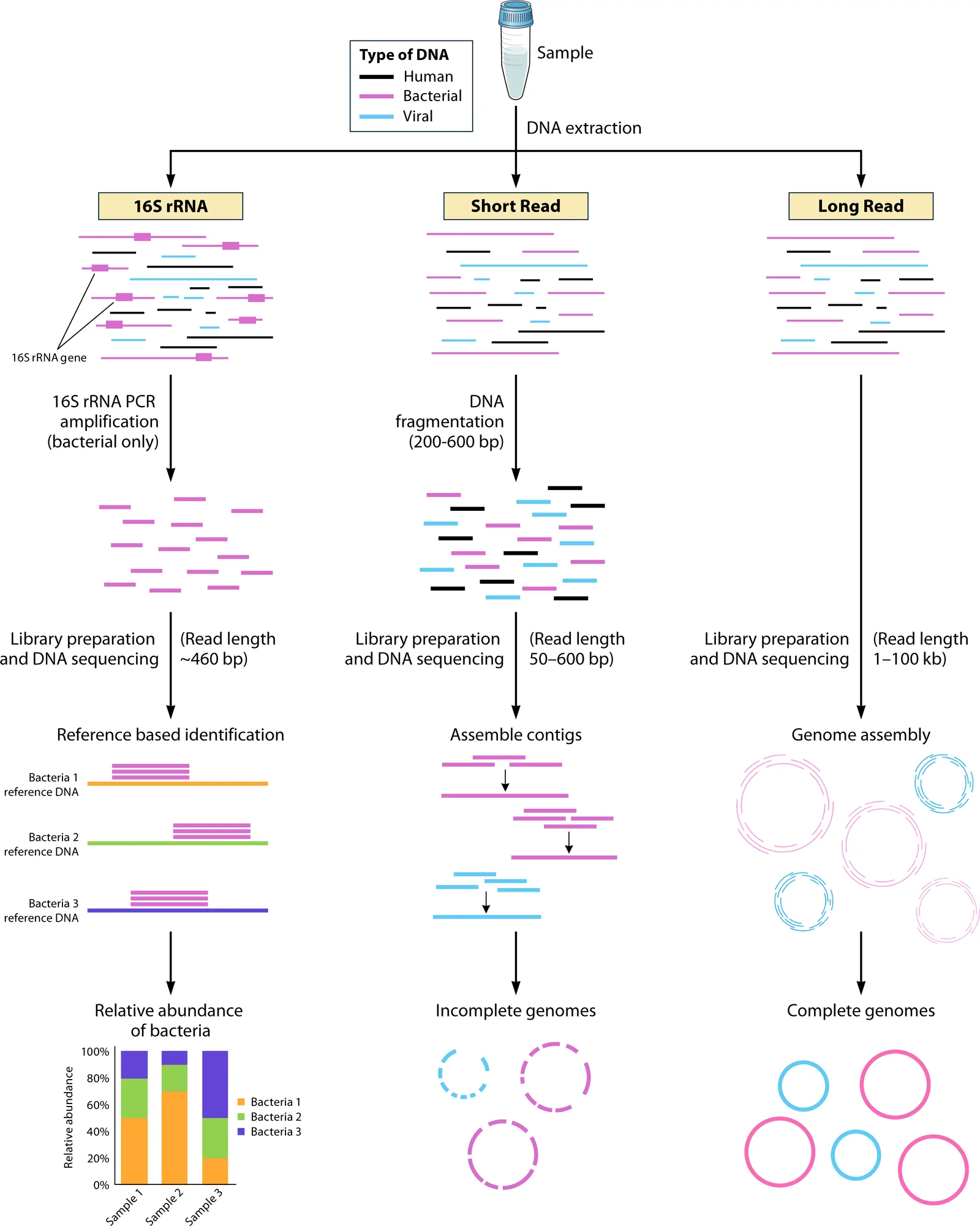
A brief overview of the differences between 16S rRNA, short-, and long-read sequencing approaches. 16S rRNA gene amplicon sequencing provides relative abundance profiles of bacteria only with limited taxonomic resolution. Short-read shotgun metagenomics enables species identification and gene detection (e.g., antimicrobial resistance genes), but genome assemblies are fragmented, and linking genes to specific hosts or plasmids is often challenging. Long-read sequencing produces contiguous assemblies that recover complete genomes, plasmids, phages, and direct linkage of AMR genes to hosts. The figure was created by the authors as an original schematic illustration using Microsoft PowerPoint (version 2605) and edited in Adobe Illustrator.

The 16S ribosomal RNA gene is found in all bacteria and contains nine hypervariable regions (V1–V9) interspersed between highly conserved regions. Universal primers are designed against the conserved regions to enable PCR amplification of the 16S rRNA gene, while sequence variation within the hypervariable regions facilitates phylogenetic classification and taxonomic identification of the bacteria in the sample (Fig. 2). As the 16S rRNA gene is absent in eukaryotic cells, sequencing is unaffected by the predominance of human host DNA in the sample. Although PCR amplification and sequencing are not quantitative, 16S rRNA sequencing provides relative abundance profiles of taxa and, through PCR enrichment, enables the detection of low-abundance and rare bacteria within the sample.

Despite its ubiquity, 16S-based profiling has its limitations. The approach lacks functional resolution, precluding the detection of mobile genetic elements that encode toxins, antibiotic resistance, or virulence traits critical to pathogenesis in CF lungs (40). The method also fails to detect viral and fungal constituents (41), thereby overlooking significant components of the airway microbiome. Taxonomic resolution is variable and often insufficient for species-level identification due to the conserved nature of sequences across clades and the variable copy number of 16S operons within and between species (42). Technical challenges such as primer bias, DNA extraction variability, and incomplete reference databases further compromise quantitative accuracy and completeness, despite attempts to quantify bacterial abundance from sequence read counts accurately (43). In addition, comparisons between different data sets become complicated when different variable regions are sequenced.

The evolution of high-throughput omics technologies has begun to address these deficits (44). Metagenomic sequencing enables taxonomic identification and genome reconstruction at strain-level resolution, offering insights into microbial dynamics and gene flow, including toxins, antibiotic resistance, and virulence determinants. Initial omics studies looked at the role of individual bacteria in disease progression (45, 46). Shotgun metagenomics provides an enhanced phylogenetic resolution over amplicon sequencing, allowing the reconstruction of individual genomes at strain-level resolution in patients to explore how strains of bacteria or viruses spread through the body and vary over time (47–50). Furthermore, separate analyses can be used to describe the metabolites present in a sample, and connecting metabolomics and shotgun metagenomics allows a deep connection between the bacteria and individual compounds that may damage the lungs (51–53).

Overall, shotgun metagenomic sequencing has contributed to an important shift in how chronic airway infection in CF is interpreted. Rather than viewing CF infections as being driven by a single dominant pathogen, the CF airway is increasingly being recognized as a complex and dynamic polymicrobial ecosystem in which microbial interactions within the airways influence disease progression and treatment response. Ecological models of the CF airway have proposed that relatively stable “climax” microbial communities can transition into more pathogenic “attack” communities during periods of exacerbation or treatment-induced disturbance (54). Metagenomic analyses of these communities have demonstrated functional differences between these ecological states, with attack-associated communities showing enrichment of genes involved in virulence and host inflammatory responses, whereas climax-associated communities appear to contain genes linked with chronic persistence, adaptive immune interactions, and reduced antibiotic susceptibility (54, 55).

Network-based analyses on metagenomic sequencing data have further demonstrated the highly interconnected nature of CF airway microbial communities and how alterations in these ecological networks are associated with disease progression (55). Within these networks, certain taxa may function as keystone species by producing metabolites, such as fermentation products, that support the growth of other opportunistic pathogens commonly associated with CF (56). In addition, metagenomic approaches have been used to infer bacterial replication rates directly within CF airway communities, providing insight into microbial activity beyond relative abundance alone (57). Metagenomics can also reveal reservoirs of antimicrobial resistance genes and provide insight into their diversity, distribution, and transmission within complex polymicrobial communities (58). This has been observed in pwCF where resistance profiles within the microbial communities evolve over time in response to antibiotic exposure, often driven by horizontal gene transfer via plasmids, phages, and other co-existing microbes (59). Together, these findings suggest that therapies, particularly antibiotics, may perturb and reshape the microbial community as a whole rather than simply targeting individual organisms. These observations highlight that CF airway disease is influenced not only by the presence of specific pathogens but also by the ecological interactions, functional dynamics, and adaptive capacity of the broader microbial community, all of which may impact disease trajectory and therapeutic outcomes.

Beyond taxonomic profiling, shotgun metagenomics can provide insight into the intraspecies population biology and genome dynamics of airway pathogens in CF. This includes characterization of the resistome (the collection of antimicrobial resistance genes), virulome (virulence-associated genes), mobilome (mobile genetic elements such as plasmids, phages, and transposons), and mutome (the accumulation of adaptive mutations within microbial populations). Longitudinal studies have shown that bacterial populations within the CF lung continuously evolve and adapt over time in response to environmental stressors, chronic inflammation, and repeated antibiotic exposure (60–63). These genetic changes can alter bacterial metabolism, motility, adherence, virulence, and antimicrobial resistance, potentially contributing to persistent infection and reduced treatment efficacy (64–67).

An important example of this is *P. aeruginosa*, a major pathogen in pwCF that can develop hypermutator phenotypes due to defects in DNA mismatch repair systems responsible for DNA proofreading (68, 69). These hypermutable strains can exhibit mutation rates up to 1,000-fold higher than wild-type strains, accelerating adaptation and the emergence of antimicrobial resistance during chronic infection (69, 70). This highlights the need for rapid culture-independent approaches capable of detecting emerging adaptive mutations and resistance determinants within clinically relevant timeframes. Rapid whole-genome sequencing approaches, such as nanopore sequencing (71) and targeted enrichment panels (72), are showing promise and may provide opportunities for earlier detection of resistance-associated mutations and more tailored antimicrobial therapy in the future.

Metagenomic sequencing can be performed using either short-read or long-read technologies, each with distinct advantages and limitations (Table 1). Short-read sequencing (e.g., Illumina and MGI platforms) produces millions of highly accurate DNA fragments typically 100–300 base pairs long. These reads are ideal for identifying known organisms and genes, but assembling them into complete genomes or linking mobile genetic elements can be difficult due to their short length (Fig. 2). In contrast, long-read sequencing (e.g., Oxford Nanopore and PacBio) generates much longer fragments, often thousands of base pairs, which can span repetitive or complex genomic regions (Fig. 2). This improves genome assembly, species-level resolution, and detection of structural variants or plasmids. However, long-read technologies typically have higher error rates and lower throughput than short-read platforms, and they require higher DNA input and quality. Increasingly, hybrid approaches that combine both methods are used to balance accuracy and completeness in airway shotgun metagenomics.

**TABLE 1:** Comparison of short-read and long-read sequencing approaches

Characteristic	Short read sequencing approaches	Long read sequencing approaches
Sequencing companies	Illumina, MGI	Oxford Nanopore, PacBio^*a*^
Read length	50–600 base pairs	1–100 kilobases
Total no. of reads per run	4 × 10^6^ to 20 × 10^10^ reads	10^5^–10^6^ reads
Genomes	Partial or near-complete assemblies	Complete genomes assembled
Plasmids	Reads need to be assembled and run through plasmid classifying tools	Miss small plasmids (73)
Viruses	Reads need to be assembled and run through viral classifying tools	Can be detected from reads or assemblies

^*a*^ PacBio, Pacific Biosciences.

Metatranscriptomics extends this framework by profiling actively transcribed genes, providing a real-time view of microbial functional activity and potential host–microbe interactions. Metatranscriptomics sequences the RNA fraction and thus captures the gene expression from those organisms, but care must be taken to avoid RNA degradation during sample preparation, and metatranscriptomes contain copious amounts of ribosomal RNA. In most cases, there is considerable overlap between shotgun metagenomics and metatranscriptomics because any unused genes are quickly lost by evolutionary selection. However, metatranscriptomics is slightly favored in rapid diagnostic approaches because it can also reveal information about the cytokine profile of the patient (44). Integration with metabolomics has furthered our understanding of host-microbial chemical exchange, linking metabolic output to disease exacerbations. We identified 24 publications or data sets in the NCBI BioProjects database that reported sputum shotgun metagenomics or metatranscriptomics from pwCF, with a mean of 32 samples and a maximum of 82 samples per study (74). A specific subset of shotgun metagenomics, known as viromics, specifically targets viral sequences. In this approach, experimental steps are employed to reduce the human and microbial load and enrich viral-like particles in the samples, while subsequent bioinformatics steps are used to identify both eukaryotic and prokaryotic viruses (bacteriophages) within the sequences (75). We only identified eight viromics samples in the databases.

**Fig 3:**
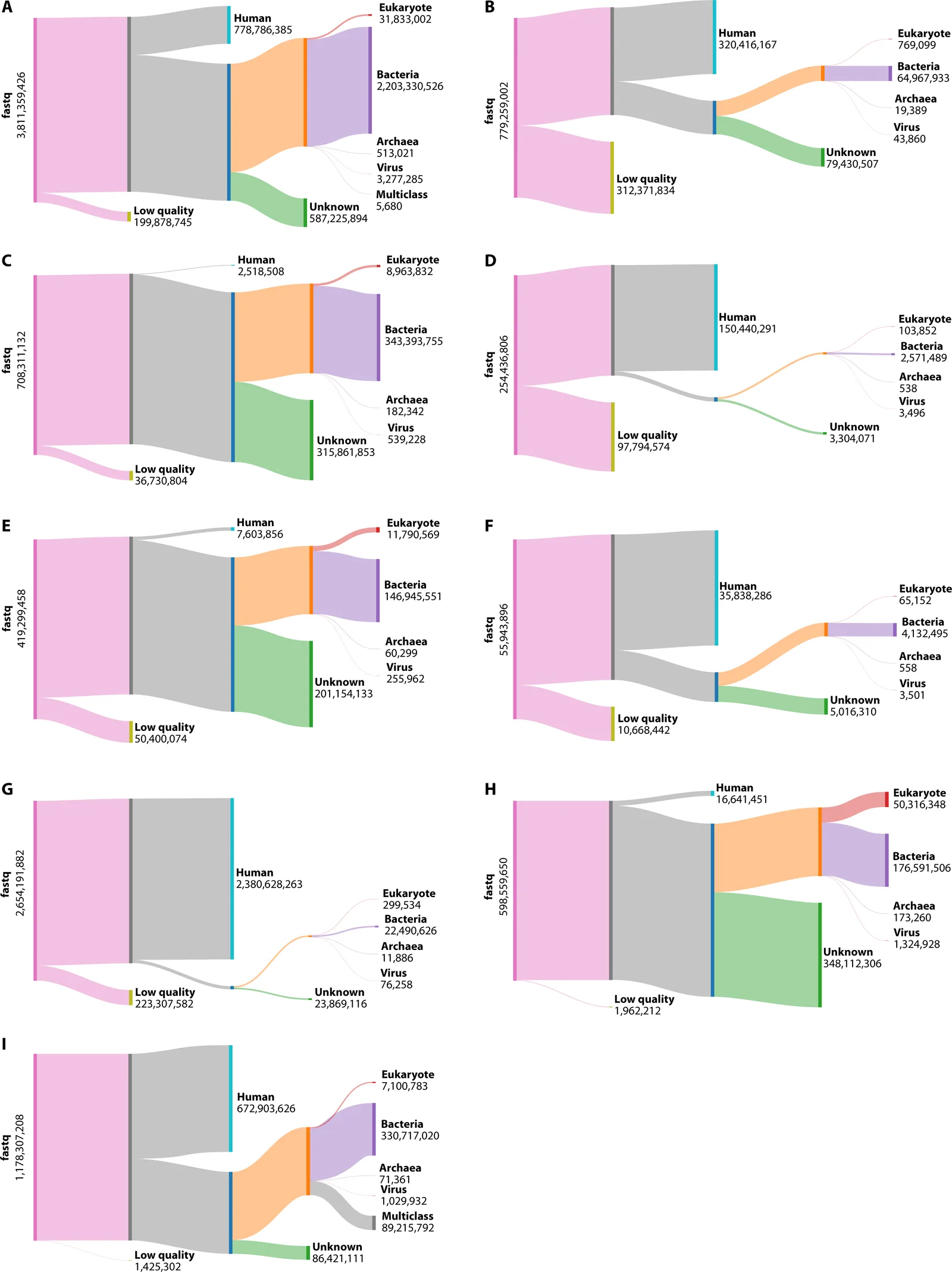
The breakdown of sputum metagenome sequences from *n* = 2,181 metagenome sequencing runs from nine different CF sputum sequencing projects. Each sample was processed and sequenced differently according to the study design; however, each data set was processed the same way using the atavide-lite pipeline (https://github.com/linsalrob/atavide_lite [76]). Sequence classifications for each project were summarized as Sankey diagrams to visualize the proportional distribution of reads across major taxonomic or sequence categories. The Sankey diagrams were generated by the authors using SankeyMATIC.com. The layout and enhancement of the figure were generated using Adobe Illustrator. (**A**) 1,409 paired-end sequencing runs from NCBI BioProject PRJEB20836 (77); (**B**) 25 paired-end sequencing runs from NCBI BioProject PRJEB32062 (47); (**C**) 71 paired-end sequencing runs from NCBI BioProject PRJNA615628 (78); (**D**) 12 paired-end sequencing runs from NCBI BioProject PRJNA644285 (79); (**E**) 141 paired-end sequencing runs from NCBI BioProject PRJNA825831 (80); (**F**) 12 paired-end sequencing runs from NCBI BioProject PRJNA839435 (81); (**G**) 61 paired-end sequencing runs from NCBI BioProject PRJNA1055940 (82); (**H**) 323 paired-end sequencing runs from NCBI BioProject PRJNA1101448 (32); and (**I**) 127 paired-end sequencing runs from EGA Study accession number EGAS50000001488 (83).

Shotgun metagenomics, metatranscriptomics, and viromics face their own obstacles. The bioinformatic analysis of these data sets is non-trivial, requiring high-throughput computing resources and expertise in microbial genomics, which are not yet widely available in routine clinical laboratories. Moreover, in addition to the high proportion of human DNA, a substantial fraction of sequencing reads remain unclassified due to the limited representation of clinically relevant taxa and viral genomes in reference databases (Fig. 3). This taxonomic ambiguity is compounded by functional annotation gaps, where identified genes frequently lack experimentally validated roles, limiting insight into virulence, resistance, or metabolic potential.

Unlike PCR diagnostics, which offer defined sensitivity and specificity, shotgun metagenomics lacks standardized thresholds for pathogen detection and clinical relevance, complicating regulatory validation and clinical decision-making. What is not known is whether the presence of a single sequence indicates that a pathogen is present (84). Variability in sample handling, nucleic acid extraction, and computational pipelines also reduces reproducibility and hinders cross-institutional comparisons. Additionally, the cost and turnaround time of metagenomic analysis remain prohibitive in many healthcare settings, especially when deep sequencing is required to capture low-abundance organisms.

Despite these challenges, advances in standardization and computational tools are facilitating the translation of metagenomics into clinical practice (85). Notably, metagenomic sequencing workflows are beginning to transition from research settings into clinically integrated services for severe respiratory infections. In the United Kingdom, routine respiratory metagenomic testing pipelines have been implemented within several intensive care units (ICUs) for rapid pathogen detection and antimicrobial resistance profiling directly from respiratory specimens (71, 86). These services have primarily been developed with oversight from diagnostic microbiology laboratories, infectious diseases specialists, and ICU teams through programs associated with tertiary referral centers, including Guy’s and St Thomas’ NHS Foundation Trust, where respiratory metagenomic sequencing has been incorporated into clinical workflows for critically ill patients (71). In addition, rapid metagenomic workflows capable of same-day pathogen detection from clinical respiratory samples continue to be developed and clinically evaluated (85, 87). Supporting this transition, the United Kingdom has also launched a national metagenomic pathogen surveillance program (88) and established the Respiratory Metagenomics Network, which aims to expand respiratory metagenomic pilot diagnostic services across up to 30 NHS sites by 2028/2029 to support faster diagnostics and more personalized antimicrobial interventions (89).

Together, these studies highlight the growing movement toward deploying metagenomic diagnostics in a clinical setting, although widespread routine implementation remains in the early stages and is currently largely limited to specialized centers. However, with continued refinement, metagenomics holds promise as a cornerstone of future infectious disease diagnostics. As such, combining culture-based methods with sequencing remains essential, not only to validate metagenomic findings but also to ensure comprehensive characterization of microbial diversity and function in clinical contexts (90). Table 2 provides an overview of the currently available pathogen identification methods used for microbial surveillance, along with their advantages and disadvantages.

**TABLE 2:** Approaches to detect microbial components in the sputum from pwCF^*a*^

Method	Target	Taxonomic assignment	Functional assignment	Time to result	Advantages	Disadvantages
Culture	Specific bacteria/fungi	Yes	No	Days to weeks	Established standard clinical diagnostic tool Isolation of live/viable pathogens Quantitative information (i.e., CFUs/mL) Antibiotic susceptibility testing	Time-consuming (slow-growing pathogens) Only targets specific pathogens Cannot provide information on overall microbial community dynamics
PCR/qPCR	Detect specific bacteria or genes	No	No	Hours	Established standard clinical diagnosis Rapid Highly specific and sensitive Can provide measure of pathogen load (using qPCR)	Limited multiplexing capacity restricts the number of targets that can be simultaneously detected in a single assay Methods need constant updating to include new and emerging resistance genes and mutations False positives Requires specialized equipment PCR inhibitors in sample may impact results
16S rRNA sequencing	16S ribosomal genes sequenced (bacteria only)	Yes	No	Days to weeks (depending on the bioinformatic support)	Broad spectrum of bacterial detection High sensitivity Culture independent	Bacteria only Lacks strain-level resolution Cannot identify AMR genes
Respiratory Pathogen ID/AMR Enrichment Panel Kit	Bacteria, virus, fungal (targets > 280 pathogens and >2,000 AMR genes)	Yes	No (except for the presence of specific AMR genes)	Days to weeks (depending on the bioinformatic support)	Reduces host DNA sequencing Rapid identification of known pathogens and AMR genes High sensitivity for targeted organisms Suitable for clinical surveillance Results easy to interpret (presence or absence) Can detect low-abundance pathogens	Only detects what the panel is designed to detect (no new, unexpected, or emerging strains detected) Does not provide complete genomes (except for SARS-CoV-2 and influenza A/B) Does not provide functional data for pathogens Need access to specialized reagents and sequencing equipment
Whole-genome sequencing (short-read)	Entire DNA	Yes	Yes	Days to weeks (depending on the bioinformatic support)	High throughput High sensitivity Rapid results Identification of non-culturable pathogens Microbiome community surveys Functional profiling AMR detection	Access to specialized non-portable sequencing equipment Sequencing run needs to be complete before data analysis can begin High cost Long turnaround time Complex DNA extraction protocol with risk of host DNA contamination Specialized bioinformatic analysis and interpretation Short reads = incomplete genomes High computational and storage requirements due to large sequencing data sets Human host contamination
Whole-genome sequencing (long-read)	Entire DNA	Yes	Yes	Days to weeks (depending on the bioinformatic support)	Rapid library preparation Real-time data analysis Long reads for full-genome coverage and assembly Option of adaptive sampling Can identify AMRs Microbiome community surveys Functional profiling Can include complete plasmid and phage sequences Portable sequencing equipment available	High cost per sample High computational and storage requirements due to large sequencing data sets Specialized bioinformatic analysis and interpretation Complex DNA extraction protocol with risk of host DNA contamination Higher error rate

^*a*^ qPCR, quantitative PCR; AMR, antimicrobial resistance; SARS-CoV-2, severe acute respiratory syndrome coronavirus 2.

## TECHNICAL CHALLENGES FOR SPUTUM METAGENOMICS

### Sample viscosity

Sputum is a highly viscous complex biological material consisting of microbial cells, mucins, and host-derived components, including extracellular DNA (91, 92). Although this complexity complicates shotgun metagenomic analysis, there are two primary experimental challenges associated with processing sputum samples. First, the high viscosity of the sputum not only makes it difficult to handle but also traps and protects the microbes present within the sample, reducing the efficiency of capturing and extracting the microbial DNA. Second, the high proportion of human host DNA within the sample can overwhelm sequencing runs, skewing the resulting microbial community profiles or missing low-abundant taxa.

Several approaches have been described in the literature to reduce sputum viscosity prior to DNA extraction. Mechanical homogenization using a sterile syringe is commonly employed to disrupt mucus plugs within the sample. Typically, sputum is mixed with sterile phosphate-buffered saline (PBS) and repeatedly passed through a syringe to disperse clumps and achieve a uniform suspension (44, 59, 93, 94). Mechanical homogenization is often supplemented with chemical treatments that target the glycoproteins responsible for mucus viscosity. These include reagents such as β-mercaptoethanol, dithiothreitol (DTT), or Remel Sputasol, which cleave the disulfide bonds cross-linking mucin glycoproteins. Reducing viscosity not only facilitates sample handling (e.g., pipetting) but also releases microbes trapped within the mucus matrix, thereby improving microbial DNA recovery (95, 96). Following this step, host DNA depletion protocols can be applied to lyse and remove extracellular DNA and human cells, including epithelial cells, macrophages, and neutrophils.

### Host DNA contamination and extracellular DNA

Human DNA can make up more than 90% of the total DNA in extracted sputum samples (45, 91, 94, 97) (Fig. 3). In pwCF, the high levels of host-derived extracellular DNA are primarily from the innate immune response to infection. During inflammation, neutrophils undergo NETosis, a process where neutrophil extracellular traps (NETs) are released that are composed of the host’s DNA and entrap invading microbes. Although human DNA sequences can be removed bioinformatically post-sequencing, excessive amounts reduce the microbial sequencing depth, potentially obscuring rare or low-abundance taxa relevant to infection and disease progression.

Several experimental approaches have been developed to deplete human host DNA before sequencing (Table 3). Saponin, a plant-derived non-ionic surfactant, can be used to selectively lyse cells containing cholesterol (98). Saponin binds to cholesterol in the lipid bilayer of human-derived cells, disrupting the cell membrane, resulting in cell lysis and DNA release (97, 99). The DNA released from lysed host cells, along with neutrophil-derived extracellular DNA, can be digested using DNase. Bacterial cells, which lack cholesterol, remain intact during this process. Following DNase treatment, the enzyme must be inactivated prior to microbial DNA extraction to prevent degradation of bacterial DNA.

**TABLE 3:** Summary of host DNA depletion and microbial enrichment strategies^*a*^

Method	Mechanism	Before or after DNA extraction	Commercial kit/software or custom protocol	References
Saponin treatment + osmotic lysis + DNase digestion	Selective lysis of cholesterol-containing eukaryotic membranes, osmotic shock of damaged cells, followed by enzymatic degradation of released host and extracellular DNA	Before	Custom protocol	(87, 97, 100)
Trypsin + detergent lysis	Selective enzymatic and detergent-based lysis of eukaryotic cells	Before	Custom protocol	(94, 101)
Osmotic lysis + enzymatic digestion (also referred to as the benzoase method in the literature)	Hypotonic lysis of host cells followed by endonuclease digestion of the released host and extracellular DNA	Before	Custom protocol	(25, 44, 51, 52, 83, 93, 94, 102)
PMA protocol	PMA crosslinks to free DNA from lysed/dead cells upon light activation, preventing its amplification or sequencing	Before	Custom protocol	(93, 94, 103)
MolYsis kit	Chaotropic lysis of host cells + endonuclease digestion of extracellular DNA	Before (kit includes DNA extraction)	Commercial kit (Molzym GmbH & Co. KG)	(93, 94, 100, 103)
HostZERO kit	Selective eukaryotic lysis + enzymatic degradation of host DNA	Before (kit includes DNA extraction)	Commercial kit (Zymo Research)	(93, 104, 105)
QIAamp DNA Host-Free Microbiome kit	Chaotropic lysis of host cells followed by enzymatic digestion	Before (kit includes DNA extraction)	Commercial kit (QIAGEN)	(93, 97, 103)
NEBNext Microbiome DNA Enrichment kit	Selective binding of methylated (host) DNA to magnetic beads; microbial DNA remains unbound	After	Commercial kit (New England Biolabs)	(94, 97, 103)
Respiratory Pathogen ID/AMR Panel	Hybridization capture of ~280 known respiratory pathogens and >2,000 AMR genes	After	Commercial kit (Illumina)	(106–108)
Adaptive sampling (MinKNOW)	Real-time rejection of human DNA reads during sequencing using a reference genome	After	Commercial software (Oxford Nanopore Technologies)	(104, 109, 110)

^*a*^ ID, identification; AMR, antimicrobial resistance; PMA, propidium monoazide; NEB, New England Biolabs.

Commercially available kits have also been developed that apply similar host depletion strategies. Most of these kits, such as HostZero, MolYsis, and QIAamp, use selective lysis of the eukaryotic cells followed by enzymatic degradation of the free-floating DNA prior to microbial DNA purification. However, the NEBNext Microbiome DNA enrichment kit uses a different approach where it captures methylated host DNA using magnetic bead-bound proteins, enabling its prior to sequencing.

To compare host depletion strategies in CF sputum for microbial shotgun metagenomics, Nelson et al. (94) compared four methods: antibody-based depletion using the NEBNext Microbiome DNA Enrichment kit, chemical lysis (101) using Trypsin-EDTA and Tween-20, a MolYsis-based method that used a chaotropic buffer to lyse human cells followed by endonuclease treatment to digest released extracellular DNA, and hypotonic lysis followed by endonuclease digestion referred to as the benzonase method (101). Nelson et al. (94) reported all approaches reduced human DNA to varying degrees, with the benzonase method being the most efficient at removing human DNA, having the greatest enrichment of microbial reads (up to a 14-fold increase) while maintaining microbial community structure and bacterial DNA yield. Furthermore, benzonase treatment enhanced antimicrobial resistance gene detection, with 67% of resistance genes identified only after depletion. Importantly, host depletion did not result in significant bacterial loss, confirming that these methods selectively remove human and extracellular DNA without damaging microbial cells. Nuclease-based treatments (benzonase and MolYsis) also increased the number of microbial taxa detected and improved sensitivity for low-abundance species.

A similar study by Kim et al. (93) investigated five host DNA depletion protocols (lyPMA, Benzonase, HostZERO, MolYsis, and QIAamp) on frozen respiratory samples. The rationale was that freezing may cause lysis of some gram-negative bacteria, and subsequent endonuclease treatment may remove the DNA from these lysed cells, leading to an underrepresentation of certain taxa and a biased metagenomic profile. The tested kits and protocols employed different approaches to host cell lysis, primarily through chaotropic lysis (HostZERO, MolYsis, and QIAamp) or osmotic lysis (lyPMA and benzonase), followed by endonuclease digestion for all methods except lyPMA. The lyPMA protocol used propidium monoazide (PMA), a photo-reactive dye that, upon light activation, cross-links to free DNA from lysed or dead cells, preventing its amplification and sequencing, while leaving DNA within intact cells unaffected. Kim et al. (93) also found that all methods effectively reduced human DNA contamination, increasing sequencing depth and improving both microbial species richness and predicted functional diversity. The efficiency of depletion varied by sample type, with MolYsis and HostZERO most effective in sputum and bronchoalveolar lavage (BAL) samples, while QIAamp and HostZERO performed best for nasal swabs.

Across all host depletion methods tested on sputum, Kim et al. reported a decrease in the relative abundance of gram-negative bacteria, including *P. aeruginosa*. Nelson et al. also observed a similar reduction in the relative abundance of *P. aeruginosa* following both benzonase and MolYsis methods (94). As *P. aeruginosa* is a well-known producer of extracellular DNA within its biofilm matrix (111), both studies hypothesized the reduction reflected removal of extracellular DNA that would otherwise inflate its representation in metagenomic profiles, rather than substantial loss of intact bacterial cells during host depletion. To investigate this further, Kim et al. cultured gram-negative (*P. aeruginosa* and *Achromobacter* spp.) and gram-positive (*S. aureus*) bacterial isolates recovered from CF sputum. Aliquots of these cultures were centrifuged to separate intact bacterial cells (pellet fraction) from extracellular material within the supernatant. Using 16S rRNA qPCR, the authors demonstrated that the gram-negative isolates contained a higher proportion of extracellular DNA within the supernatant compared to the gram-positive isolates. Based on this, the authors concluded that the observed reduction in the relative abundance of gram-negative species following host depletion was likely due to degradation of extracellular DNA rather than the loss of viable bacterial cells.

To test the effect freezing had on the viability of these gram-negative and gram-positive isolates, Kim et al. performed colony-forming unit (CFU) tests. They reported that freezing did not impact the viability of the gram-positive bacterial isolates but reduced the viability of gram-negative isolates, an effect largely mitigated through the addition of a cryoprotectant (glycerol). Among the host depletion methods, MolYsis had the greatest impact on cell viability of the frozen gram-positive isolates, whereas QIAamp had the least effect in both cryopreserved and non-cryopreserved gram-negative isolates. Overall, Kim et al. concluded that, while host depletion and storage conditions may introduce bias in metagenomic profiling, omitting host depletion will severely underestimate microbial diversity in respiratory samples, particularly sputum and BAL, due to overwhelming host DNA masking the true microbial signal.

Despite best efforts to remove the human host DNA prior to sequencing through experimental depletion methods, residual human DNA will still be present post-sequencing (Fig. 3). Most of these sequences can be removed using bioinformatic tools that map reads to the human genomes to identify and filter out human-derived DNA (112, 113). The removal of human reads during bioinformatic processing therefore represents an essential final step in data processing, particularly prior to deposition of data sets in public repository databases, where protecting participant privacy is an ethical requirement.

Beyond physical and enzymatic host depletion strategies, targeted enrichment approaches provide an alternative means of reducing host DNA interference. The Illumina Respiratory Pathogen ID/AMR Enrichment Panel detects approximately 280 known respiratory pathogens and 2,000 AMR genes (106–108, 114). Although host DNA remains present in the sample, the panel reduces human DNA interference by using hybridization probes to enrich microbial DNA and cDNA (converted from RNA) extracted from sputum. This enables sequencing of only the targeted regions on the Illumina platform while effectively excluding host DNA. This approach efficiently identifies the presence or absence of known pathogens and resistance genes. Although full-genome coverage is achievable for some viruses (e.g., SARS-CoV-2, influenza A/B), most organisms are detected through short, predefined genomic regions. This limitation reduces taxonomic resolution and restricts the ability to detect novel strains or genes. Therefore, while ideal for rapid screening of known targets of interest, it does not capture the entire microbial community that whole-genome sequencing provides. This includes commensals, rare taxa, and novel resistance mechanisms that are important in pwCF, especially in the evolving post-modulator era.

An emerging alternative to combat the high levels of host DNA contamination in metagenomic samples is adaptive sampling on the Oxford Nanopore sequencing platform. This real-time sequencing technique enables the enrichment or depletion of specific DNA molecules within a mixed sample as sequencing occurs (115). As each DNA strand begins to pass through a nanopore, its sequence is compared to a reference genome/sequence (i.e., the human genome), and the sequencing software determines whether to continue sequencing or reject the DNA molecule in favor of another (i.e., microbial DNA). By rejecting human DNA in real time, adaptive sampling can enrich for bacterial and other microbial DNA without the need for host depletion strategies. Although still an emerging methodology (110), this technique has been applied to respiratory samples, demonstrating its potential future use in microbial genome profiling in samples with high host DNA contamination (109).

### Upper airway and technical contamination of samples

The potential contribution of upper airway microbial communities to lower airway samples, particularly in expectorated sputum, is an important consideration in CF metagenomic studies. Contamination may occur during sample collection as sputum passes through the oropharynx and oral cavity, introducing upper airway taxa into the specimen. Although BAL samples are less susceptible to this effect, some degree of upper airway carryover may still occur via cross-contamination from the bronchoscope during passage through the upper airway (116). Strategies to mitigate upper airway contamination include oral rinsing prior to sample collection (59), paired sampling of saliva and sputum (117), and protected bronchoscopy techniques (116, 118).

In addition to upper airway contamination, metagenomic sequencing must also consider technical sources of contamination introduced during specimen collection, DNA extraction, and library preparation. In low-biomass samples, such as BAL, introduced background microbial DNA may contribute to the final observed microbial profile. Therefore, the inclusion of appropriate negative controls such as sequencing the blank “sterile” saline lavage fluid, laboratory reagents, and extraction kit blanks used in the extraction process are important in identifying and removing background microbial signals (119). Contaminating sequences can subsequently be removed during bioinformatic processing through comparison with sequenced negative controls or the use of dedicated decontamination tools designed to identify and filter background contaminants (112, 119, 120). However, taxa traditionally associated with the oral cavity or upper airways should not always be assumed to represent contamination, as microaspiration and the anatomical connection between the oral cavity and lungs may contribute to their presence in lower respiratory samples (46, 121). Although there is some overlap between sputum and saliva microbiomes, the impact of oral contamination on sputum samples has been shown to be minimal, supporting its continued use in CF microbiome studies (117).

### Hard-to-lyse bacteria

Nontuberculous mycobacteria (NTM) can cause chronic pulmonary infections in pwCF and represent a significant clinical challenge for health care professionals (122, 123). Conventional culture-based identification of this pathogen remains difficult due to their slow growth, often requiring more than 6 weeks before a positive result is reported (122). Therefore, there is growing interest in the use of metagenomics to expedite the detection of NTM for use in a clinical setting (124–127). However, the recovery of NTM in sequencing-based approaches, especially when utilizing whole-genome metagenomics, remains challenging and is often dependent on DNA extraction efficiency (128).

Mycobacteria have a tough, hydrophobic, lipid-rich cell wall containing mycolic acids that forms a robust barrier to lysis, often resulting in their underrepresentation in sequencing data sets if not adequately disrupted (128–130). Mechanical lysis methods, such as bead beating, are commonly used to improve DNA yield from these organisms; however, excessive processing may lead to DNA fragmentation, which is suboptimal for long-read sequencing applications where building complete bacterial genomes is of interest (131, 132). Enzymatic lysis strategies optimized for hard-to-lyse gram-positive bacteria, including treatment with lysozyme to degrade peptidoglycan components of the cell wall, are also frequently incorporated to enhance extraction efficiency (131, 133). These methodological considerations are therefore critical for ensuring optimal detection and characterization of mycobacteria and other hard-to-lyse bacteria in CF airway microbiome studies.

### Sputum reduction post-modulator therapy

Sputum has historically been the most widely used sample type for investigating the airway microbiome in pwCF, as it provides a non-invasive means of assessing lower airway microbial composition. Before the introduction of CFTR modulator therapy, many pwCF routinely produced large volumes of sputum, enabling repeated, minimally invasive sampling compared with more invasive procedures such as BAL. Therefore, sputum became the standard specimen submitted for microbiological testing and research aimed at identifying airway microbial communities. However, since the introduction of triple modulator therapies, sputum production has markedly declined, with some individuals becoming non-productive as early as 14 weeks after initiating treatment (25). This reduction in sputum availability presents a growing challenge for airway microbiome monitoring. Alternative sampling approaches, including oropharyngeal, nasal, and cough swabs, induced sputum, and BAL, are now being explored (25, 30, 32, 134). However, these samples differ in microbial composition, depth of airway representation, and ease of collection. For example, oropharyngeal, nasal, and cough swabs are minimally invasive and easy to collect but primarily capture microbial communities of the upper airway, which may not accurately reflect lower airway infections (134–138). In contrast, BAL samples are considered more representative of the lower airway environment but are invasive and therefore less suitable for routine or longitudinal sampling (138, 139). Induced sputum is considered the closest approximation to expectorated sputum and may provide a more representative assessment of lower airway microbial communities while remaining a relatively non-invasive sampling approach (140). Therefore, careful consideration is required when interpreting and comparing microbiome data generated from different sample types, particularly in studies spanning the pre- and post-modulator eras, as sampling methodology may influence observed microbial profiles.

## IMPACT OF METAGENOMIC SEQUENCING ON CLINICAL DECISION-MAKING

Many studies have used whole-genome sequencing to characterize the respiratory microbiome in pwCF within a research setting. However, the key challenge now lies in determining how this data can be integrated into clinical workflows and translated into actionable decisions that ultimately improve health outcomes for individuals. Wu and Zinter (2025) outlined a practical roadmap for translating respiratory metagenomics into routine clinical care and highlighted four major hurdles that should be addressed before widespread adoption of metagenomics is implemented within a clinical setting (141).

First, metagenomic sequencing must clearly demonstrate improved diagnostic yield compared with traditional culture-based and antigen-based methods (141). Numerous studies have demonstrated the enhanced microbial identification potential using a metagenomic approach (45, 59, 105, 142). A compelling demonstration of this is shown in a case study by Cobián Güemes et al., in which a CF rapid response strategy was used to investigate potential reasons for a fatal pulmonary exacerbation in an individual with CF (142). This rapid response strategy utilized viromics, metagenomics, metatranscriptomics, and metabolomics to rapidly characterize active microbial and viral communities during acute exacerbations. While standard culture-based diagnostics identified typical CF-associated pathogens including *P. aeruginosa* and *S. maltophilia*, multi-omics (metagenomics, metatranscriptomics, and metabolomics) was also applied to further investigate the individual’s fast decline in lung function. Metatranscriptomic analyses of longitudinal sputum samples collected prior to the individual’s death identified a shigatoxigenic *Escherichia coli*, a pathogen not routinely screened for in CF respiratory samples. Beyond enabling the identification of an organism that would likely have remained undetected using standard CF culture panels, metagenomic sequencing demonstrated potential clinical utility by providing information that could shape antimicrobial selection. In this case, identification of toxin-associated virulence genes may have informed antibiotic selection, as some antibiotics can induce bacterial stress responses associated with increased Shiga toxin release, whereas others are less likely to do so (143–147). In addition, adjunctive therapeutic strategies, such as the administration of neutralizing antibodies targeting the Shiga toxin, could also have been considered (142). Although sequencing results were not used to alter the treatment of the individual, this case highlights several clinically relevant advantages of whole-genome sequencing and metatranscriptomics.

Second, metagenomic workflows must be shown to be technically and logistically feasible in real time (141). This includes rapid sample processing, DNA sequencing, bioinformatics, clinical interpretation, and reporting within the tight timeframes required for acute respiratory care. Recent advances in sequencing platforms, streamlined library preparation, and automated bioinformatic pipelines have substantially reduced turnaround times, making near–real-time respiratory metagenomics increasingly achievable (71, 85, 86, 142, 148). Nevertheless, successful clinical implementation will require robust laboratory infrastructure, standardized and validated analytical pipelines, and clear reporting frameworks that can be readily interpreted by clinicians.

Third, it is important to demonstrate how whole-genome sequencing results influence clinical decision-making (141). This includes whether sequencing results lead to changes in antimicrobial selection, reduce unnecessary antibiotic escalation, or enable antibiotic de-escalation through improved pathogen identification, detection of antimicrobial resistance genes, and recognition of viral-driven infections. Time pressure and diagnostic uncertainty often lead clinicians to prescribe broad-spectrum antibiotics empirically with a “just in case” approach (149, 150). By enabling direct pathogen identification, detection of antimicrobial resistance genes, whole-genome sequencing has the potential to reduce diagnostic uncertainty and limit inappropriate antibiotic use. Emerging evidence suggests that sequencing can influence clinical decision-making in respiratory infections, with several studies reporting sequencing-guided modifications to treatment strategies (71, 151–153). For example, in a cohort of 400 individuals with suspected lower respiratory tract infections, Lai et al. (151) reported that sequencing-guided results led to modification of antimicrobial therapy in more than half of the confirmed cases, including antimicrobial adjustment, de-escalation, and escalation (151). Overall, 60.8% of individuals showed clinical improvement following the sequencing-guided modification of antimicrobial therapy.

However, the clinical interpretation of sequencing data remains complex, particularly in respiratory samples where diverse microbial communities are present. Metagenomic approaches can detect a broad range of organisms, including both pathogens and commensal airway taxa present at varying relative abundances, making it challenging to identify the organisms responsible for infection. In this context, the detection of additional organisms through DNA sequencing may inadvertently have a negative influence on diagnostic decisions, potentially leading to unnecessary or overly broad antimicrobial use. Furthermore, targeted antimicrobial therapy against specific organisms may alter microbial community structure and create ecological niches that facilitate the expansion of other opportunistic pathogens that were previously suppressed. While these findings indicate that sequencing can influence clinical decision-making, whether such changes consistently translate into improved patient outcomes remains an important unanswered question.

This leads to the fourth, and most important consideration: whether access to real-time whole-genome metagenomic data results in measurable improvements in clinical outcomes, including faster recovery, reduced antimicrobial exposure, and fewer complications (141). Studies in severe respiratory infections, such as pneumonia, suggest that metagenomic sequencing can facilitate earlier targeted therapy and accelerate clinical improvement compared with conventional culture-based microbiological testing alone (151, 152, 154). Whether these benefits translate to the complex and chronic infection landscape of pwCF remains uncertain and represents an important area for future research. Only by meeting these four criteria can next-generation sequencing transition from a promising diagnostic innovation to a routinely actionable tool in respiratory medicine.

Currently, there is insufficient evidence to support metagenomics as a standalone diagnostic technique for respiratory CF specimens. Therefore, it should be used as a complementary tool alongside established conventional microbiological testing (culturing and PCR detection) to support clinical diagnostics and therapeutic decision-making. However, the greatest clinical utility of metagenomics at this stage may be in complex cases involving difficult-to-culture or unidentified organisms that are not detected through routine culture-based tests. It may also be useful in patients who fail to respond to treatment, where additional investigation, such as assessment of resistance genes, could help optimize therapeutic strategies. As sequencing technologies continue to advance, and more standardized protocols for specimen collection, processing, sequencing, and bioinformatic analysis are developed, metagenomics may become increasingly suitable as an independent diagnostic approach for respiratory medicine and other microbially driven diseases in the future. Only by meeting these criteria can next-generation sequencing transition from a promising diagnostic innovation to a routinely actionable tool in respiratory medicine.

## LOGISTICAL CHALLENGES FOR CLINICAL METAGENOMICS

While metagenomics shows strong potential for rapid and accurate pathogen identification and is beginning to be adopted into the clinical setting (71, 85, 148), widespread implementation remains limited. We have identified six primary barriers that continue to challenge the adoption of metagenomics into the broader clinical setting.

### Cost per sample

Deep-sequencing shotgun metagenomics is usually necessary to overcome the abundant human DNA in the sample, the diversity of the bacteria, and the limits of viral DNA. Even with the latest generation of high-throughput sequencing platforms, generating sample libraries and sequencing still costs approximately $250 AUD per sample, resulting in approximately 4 million reads per sample. However, improved human depletion, adaptive sampling, and long-read sequencing are mitigating this.

### Parallelization

The high-throughput sequencers achieve cost reduction by economies of scale. In a diagnostic situation with only a few patients, weeks may elapse before accumulating enough samples to run the sequencing machines, eliminating the possibility of rapid diagnostic sequencing. In contrast, long-read sequencing with the Oxford Nanopore MinION usually involves multiplexing four to six samples, facilitating day-to-day operations.

### Sample and library preparations

While not excessively onerous, the typical sample and library preparation protocols take approximately 12–24 hours, depending on how many samples are being processed (142). Preprocessing the sputum takes approximately 2 hours, DNA extraction takes 90 minutes, and library preparation takes about eight hours. Without automation, these processes cannot be supported by the clinical laboratory. In addition, there are currently no widely approved, standardized, automated DNA extraction protocols that include appropriate quality control measures. However, with optimized host DNA depletion and sample preparation strategies as discussed above, combined with advancements in rapid library preparation and real-time sequencing technologies, it is now possible to complete the process in as little as 2–4 hours (71, 85, 86, 148).

### Bioinformatics analysis

Sequencing data generated require specialized bioinformatic analysis, typically performed in a Linux-based analysis environment by trained bioinformaticians. Such expertise is not commonly available in routine clinical laboratories, creating a significant barrier to the integration of sequencing into diagnostic workflows. Additionally, the analysis of the sequencing data is computationally intensive, requiring access to high-performance computing infrastructure. The outputs generated from the data analysis (i.e., long lists of bacterial taxa, genes, putative functions, and possible antibiotic resistance markers) are complex and not easily and rapidly interpreted by clinicians into clinically actionable information. Most bioinformatic analysis tools are designed for research applications, focusing on exploratory data from an environment rather than reporting on the information that is clinically relevant. In most cases, the delay from sample collection to bioinformatics analysis has limited the clinical use of sequencing data. It is usually more expeditious to rely on traditional diagnostic approaches that, *in most cases,* lead to clinical improvement and/or the eradication of the infectious pathogen. Addressing this gap will be crucial for translating sequencing-based approaches into tools that can meaningfully predict future clinical outcomes in cystic fibrosis and other chronic respiratory diseases.

### Prognostication

The markers for future adverse events or clinical deterioration may already be present in the sequencing data generated today. This includes the emergence or persistence of pathogens, microbial community shifts, or emergence of resistance genes. However, accurately identifying these signals amidst a complex microbial landscape remains a major challenge. Overlooking these markers can have critical consequences for treatment decisions or adverse patient outcomes and may also carry medicolegal implications related to delayed or inadequate care. There are many taxa detected in sequencing data, many of which have uncertain clinical relevance, and their role in disease progression is often poorly understood. For example, the presence of a pathogen in one patient might be associated with worsening lung function or exacerbations, while in another patient, the same organism might be benign or even part of a relatively stable microbiome. This highlights the inadequacy of a “one size fits all” model in microbiome interpretation and risk prediction. Additionally, host factors, including genotype, immune status, prior antibiotic exposure, and underlying lung pathology, can all modulate the clinical impact of a given microbial profile (155–157). As such, effective prognostication may require patient-specific microbiome baselines and longitudinal data to detect meaningful deviations over time, rather than relying solely on cross-sectional comparisons.

### Data storage and ethical considerations

Metagenomic sequencing generates large data sets that require substantial computational infrastructure and long-term storage capacity. As previously discussed, residual human DNA may still remain detectable in metagenomic data sets despite laboratory-based host depletion and bioinformatic filtering strategies. Therefore, the generation, storage, sharing, and analysis of these human microbiome data sets require careful long-term governance to protect participant privacy and ensure appropriate management of potentially identifiable genetic information. As clinical metagenomics becomes more widely adopted, robust governance frameworks and ethical guidelines will be essential, particularly when collecting and managing genomic data from communities that may be disproportionately affected by misuse or unintended disclosure of genetic information (158–160).

## CONCLUSION

Culture-independent analysis, such as DNA sequencing, has revolutionized our understanding of the microbial dynamics in CF, unlocking the hidden diversity traditional culturing methods fail to detect. DNA sequencing enables a comprehensive exploration of the microbial communities within the CF lung, the interactions between these microbial communities, and the response of the CF lung microbiome to differing treatments and interventions. In doing so, DNA sequencing can pave the way for innovative applications in diagnostics and personalized medicine. Rapid analysis of CF lung microbiome data may enable timely and appropriate clinical intervention, preventing chronic infection and colonization. However, it is also clear that the translation of DNA-sequencing-based CF microbial research into clinical application remains in its infancy. Further research is required to define optimal airway specimen collection strategies and standardize the generation, processing, analysis, and interpretation of microbiome data in the CFTR modulator era.

The treatment of CF is at a pivotal moment, with CFTR modulator therapies potentially altering the relentlessly progressive inflammation and overwhelming infections that irreversibly alter the anatomy and function of the CF lung. Understanding the effects of CFTR modulators on the CF lung microbiome will involve analyzing pre- and post-modulator samples and tracking changes longitudinally over time (25, 28, 32, 161). In addition, differences between individuals commencing modulators later in life compared with those treated from early childhood may further influence airway microbial composition and long-term disease progression. The marked reduction in spontaneous sputum production following modulator therapy also highlights the need to evaluate alternative minimally invasive sampling approaches for longitudinal airway monitoring. As respiratory samples from pwCF become less readily available in the modulator era, maximizing the biological and clinical information obtained from each specimen will become increasingly important. In this setting, culture-independent sequencing approaches may provide substantial advantages by enabling comprehensive microbial, functional, and resistance profiling from limited sample volumes. Capturing these microbial changes will enable researchers and clinicians to gain deeper insight into how lung infection, colonization, inflammation, and damage occur in CF, thereby highlighting gaps in evidence and areas to focus future research and therapeutic developments for pwCF.

Collaborative, multi-disciplinary teams, including microbiologists, bioinformaticians, and clinical experts, are making strides in developing the next generation of clinical reports to harness metagenomics, metabolomics, and other omics data and bring those technologies to the clinic (71, 86, 142). The emergence and advances in portable, real-time sequencing devices, especially the Oxford Nanopore sequencing technologies, are beginning to enable sequencing-based diagnoses in the clinical laboratory (71, 85, 148). As these technologies continue to demonstrate improvements in patient care and outcomes, clinical demand will likely drive the innovation required to bring sequencing to the forefront of diagnostic microbiology. Beyond specimen collection, the continued generation of large-scale metagenomic data sets presents additional opportunities for machine learning and artificial intelligence approaches to identify microbial signatures, resistance patterns, and predictive biomarkers (83). These approaches may ultimately assist in the interpretation of complex microbiome data and support more personalized treatment decision-making in CF and other microbially driven infections in the future.
